# Trends in body mass index and energy intake with and without biomarker calibration in the USA and Japanese National Nutrition Surveys

**DOI:** 10.1017/jns.2025.10069

**Published:** 2026-01-21

**Authors:** Yumiko Inoue, Daiki Watanabe, Motohiko Miyachi

**Affiliations:** 1 Graduate School of Sport Sciences, Waseda University, Tokorozawa, Saitama, Japan; 2 National Institute of Biomedical Innovation, National Institutes of Biomedical Innovation, Health and Nutrition, Ibaraki, Osaka, Japan; 3 Faculty of Sport Sciences, Waseda University, Tokorozawa, Saitama, Japan

**Keywords:** Calibration approach, Energy intake, Physical activity, Self-reported dietary Assessment

## Abstract

In the USA and Japan, body mass index (BMI) has increased over the last several decades, whereas energy intake (EI) has decreased. However, self-reported EI data may show systematic errors. Using the calibration approach for attenuating the systematic error of self-reported EI, we aimed to compare trends in BMI and EI with and without calibration in adults from the USA and Japan. This cross-sectional study included 38,370 Americans evaluated in the National Health and Nutrition Examination Survey 2003–2018, and 200,629 Japanese evaluated in national nutrition surveys in Japan 1995–2019. EI was estimated using at least 1 day of 24-h diet recalls for Americans and 1 day of household-based dietary records for Japanese. The calibrated EI was calculated using a previously developed equation based on total energy expenditure (TEE) measured by doubly labelled water method. Using data from a review, uncalibrated EI was −20.2% and calibrated EI was −4.1% compared to the TEE; the calibration approach attenuated EI underestimation. In the USA, uncalibrated EI decreased (annual percentage change [APC]: −0.24%), but calibrated EI and BMI increased (calibrated EI, APC: 0.04%; BMI, APC: 0.32%). In Japan, the decrease was smaller for the calibrated EI than for the uncalibrated EI (uncalibrated EI, APC: −0.23%; calibrated EI, APC: −0.04%). Uncalibrated EI decreased and BMI increased in the USA and Japan, and calibrated EI increased in the USA and decreased slowly in Japan. Calibration may attenuate systematic bias in dietary assessments and facilitate the effective use of dietary data.

## Introduction

The coexistence of undernutrition and overnutrition (overweight and obesity), known as the double burden of malnutrition, is a global problem affecting many countries, including the USA and Japan.^(1)^ This phenomenon poses a threat to public health by increasing the risk of non-communicable diseases, childbirth complications, and mortality.^(2–5)^ Body mass reflects long-term changes resulting from the dynamic equilibrium between dietary energy intake (EI) and total energy expenditure (TEE).^(6)^ Physical activity-related energy expenditure is the most variable component of TEE.^(6)^ The USA and Japan are both high-income countries that have experienced substantial economic development, but they have had contrasting historical changes in body mass; over the past several decades, the prevalence of obesity has increased markedly in the USA, whereas changes in body mass have been more gradual in Japan. Thus, a comparison of these two contrasting countries may provide insights into long-term changes in EI or energy balance.

Data from national surveys conducted in both Japan and the USA have reported a decreasing trend in EI assessed using 24 h-diet recalls (24HR) and dietary records (DR) over the past few decades.^(7,8)^ Such self-reported dietary data are subject to systematic errors related to individual characteristics, such as body mass index (BMI) and age; for example, individuals with higher BMI are known to underreport their EI.^(9–11)^ The calibration approach, using equations derived from biomarkers, can correct for the systematic underreporting associated with individual characteristics such as high BMI and general measurement errors inherent in dietary surveys.^(12)^ Dietary assessments using biomarkers, as recommended in nutritional epidemiology guidelines^(13)^, are expensive. In contrast, this calibration approach can partially attenuate systematic errors without requiring the collection of additional new data, making it a cost-effective method. Previous studies have reported that calibrated EI is associated with cardiovascular disease, diabetes, cancer, and all-cause mortality, whereas uncalibrated EI is not.^(14–16)^ This approach enables examination of the relationship between EI and body mass at the population level with attenuated systematic errors due to dietary assessment, unlike in previous studies that did not consider such errors.^(7,8)^

Thus, this study aimed 1) to evaluate the validity of a previously developed and reported EI calibration equation (9) using literature data including TEE measured by the doubly labelled water (DLW) method as a reference, to determine its applicability to other populations, and 2) examine trends in EI, measured with and without the calibration approach, and BMI using national survey data from the USA (2003–2004 to 2017–2018) and Japan (1995–2019). We also evaluated the relationship between EI and physical activity in the Japanese population to better account for overall energy balance. Our hypothesis was that uncalibrated EI would decrease and BMI would increase in both American and Japanese populations, as shown in previous studies.^(7,8)^ In contrast, we hypothesised that the calibrated EI would show an increasing trend over the past few decades by attenuating the estimated underestimation of EI in self-reported dietary assessment of individual characteristics such as BMI.

## Experimental methods

### Study design and population

**Figure 1.**
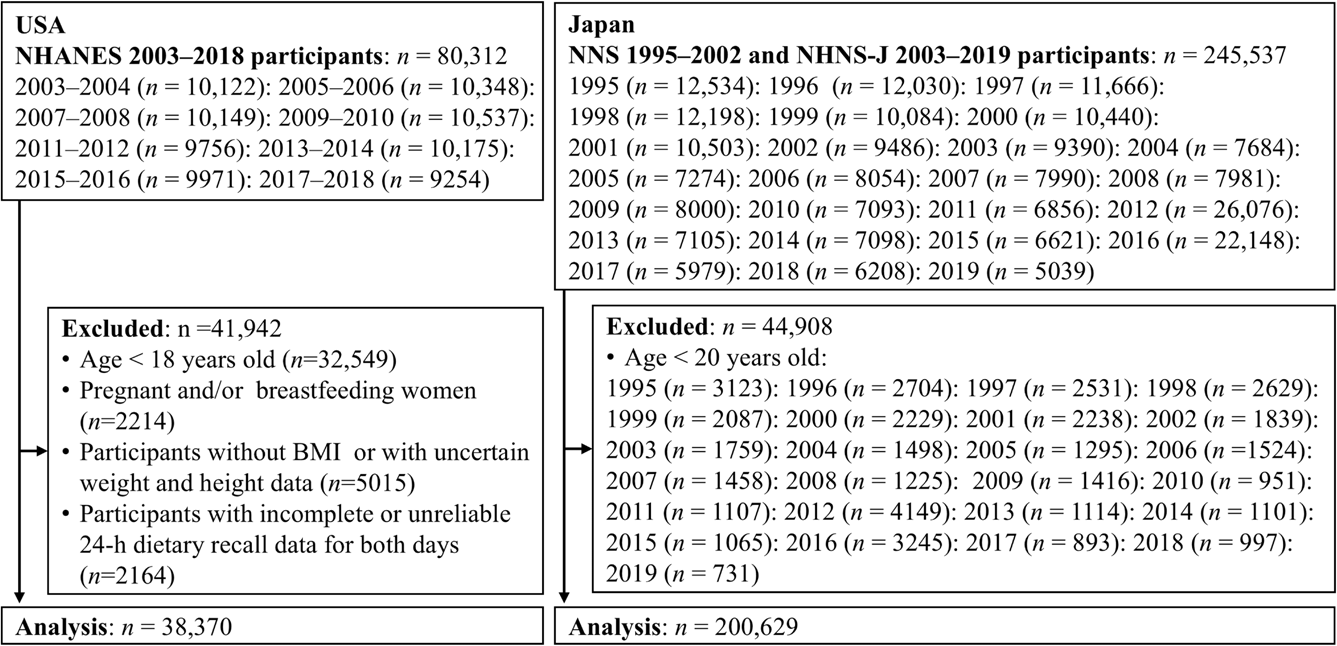
Participant flow diagram for the analysis using data from national surveys for USA and Japan. NHANES, National Health and Nutrition Examination Survey; NNS-J, National Nutrition Survey Japan; NHNS-J, National Health and Nutrition Survey Japan; BMI, Body mass index.

This cross-sectional study used data from the National Health and Nutrition Examination Survey (NHANES) in the USA, the National Nutrition Survey of Japan (NNS-J; conducted until 2002) and the National Health and Nutrition Survey of Japan (NHNS-J; conducted since 2003) in Japan. NHANES is a recurring cross-sectional survey representative of the non-institutionalised American citizen population and is conducted by the National Centers of Health and Statistics (NCHS), part of the Center for Disease Control and Prevention. Details of the survey procedure have been previously reported.^(17)^ The survey uses a complex multi-stage probability sampling design to reflect the American population and is conducted through face-to-face household interviews along with assessments in a mobile examination centre (MEC). We electronically extracted data from eight two-year cycles from 2003 to 2018 that were available on a website maintained by the NCHS.^(18)^ A total of 80,312 individuals participated in the NHANES 2003–2018, excluding those younger than 18 years (*n* = 32,549), pregnant and/or lactating women (*n* = 2,214), those for whom accurate height and weight data were not available (*n* = 5,015), those who provided dietary information deemed unreliable by trained NHANES interviewers, and those with missing 24HR data (*n* = 2,164). Finally, 38,370 participants (women: *n* = 19,076; men: *n* = 19,294) were included in the analysis (Figure 1).

The NNS-J and NHNS-J are nationally representative, recurring, and cross-sectional surveys conducted by local health centres under the supervision of the Japanese Ministry of Health, Labour, and Welfare. Details of the NNS-J and NHNS-J survey procedures have been described previously.^(8,19,20)^ Briefly, a sample was selected using a two-stage cluster sampling scheme to reflect the Japanese population, although the 2012 and 2016 expansion surveys were conducted using a stratified one-stage cluster sampling. These surveys were conducted from 25 October to 7 December in 2012, from October to November in 2016, and in November of each year. These surveys collected data on health status, dietary intake, and lifestyle habits of individuals. We obtained data stratified by sex and age (20–29, 30–39, 40–49, 50–59, 60–69, and ≥70 years) from aggregate reports available electronically on the official website of the Ministry of Health, Labour, and Welfare.^(21)^ Of the 245,537 participants in the 1995–2019 NNS-J and NHNS-J, those aged <20 years (*n* = 44,908) were excluded. Thus, we included 200,629 individuals (women: *n* = 110,780, men: *n* = 89,849) in the analysis (Figure 1). Body mass data excluded pregnant and breastfeeding women, whereas dietary intake analyses used aggregated data that included these individuals, who represented about 1% of the survey population. Because we relied on aggregated data, we could not exclude them; however, given their small proportion, this discrepancy is considered to have had only a minimal impact on EI.

### Ethical approval

The NHANES, NNS-J, and NHNS-J surveys were conducted in accordance with the tenets of the Declaration of Helsinki, and verbal informed consent was obtained from all participants. In accordance with the ‘Ethical Guidelines for Epidemiological Research’ issued by the Ministry of Education, Culture, Sports, Science and Technology and the Ministry of Health, Labour and Welfare, we determined that an ethical review was not required because we used only annual reports and summary table data, which were anonymised and did not contain personally identifiable information.

### Dietary assessment

In the NHANES, dietary intake was assessed using a 24HR administered by trained interviewers. The US Department of Agriculture (USDA) automated Multiple-Pass Method, an automated 5-step multiple-pass approach, was used to collect 24HR data.^(22)^ Participants were asked to report all foods and beverages consumed between midnight and midnight for the previous 24 h. The EI was calculated using the USDA Food and Nutrition Database for Dietary Studies for all reported food and beverage intakes.^(23)^ In previous studies, the accuracy of a single 24HR estimate was reported to have a correlation coefficient of *r* = 0.5 to 0.6 with the mean 6-month EI of individuals with overweight and obesity.^(24)^ EI was calculated from at least one 24HR.

In the NNS-J and NHNS-J, dietary intake was assessed using a one-day semi-weighed household DR, excluding travel and holidays.^(25)^ Before conducting the dietary survey, participants were instructed to complete the DR by well-trained staff (primarily dietitians). Household members who usually prepared meals were asked to weigh all foods and beverages consumed by household members and assign an approximate proportion of each meal item to each household member. If data were missing or illogical, workers visited the homes to verify the food and beverage portions listed in the food record forms. EI was calculated from the weight of the foods and beverages consumed by individuals and from food composition tables. The food composition tables were the latest Japanese Standard Tables of Food Composition available at the time.^(26–30)^

### Calculation of calibrated EI

Since EIs calculated from self-reported dietary surveys are underreported, we calculated a calibrated EI using equations previously developed for Japanese older adults.^(9)^ This equation was developed to reduce systematic errors in the estimation of the EI-assessed food frequency questionnaire (FFQ). A stepwise multiple regression analysis was performed using TEE, which was measured using the DLW method, as the dependent variable. Age, sex, BMI, and EI estimated using the FFQ were included as independent variables. The coefficient of determination (R^2^) was 0.36. The following equations are used in the model:
(1)

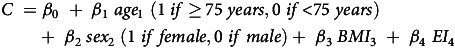

where *C* denotes the calibrated EI. The intercept (β0) of the equation was 1384.92 kcal. The coefficients of binary variables were −166.98 kcal for age (β1) and −354.72 kcal for sex (β2). The coefficients of the continuous variables were 25.55 kcal (kg/m^2^) for BMI (β3) and 0.24 kcal (kcal/day) for EI (β4). The product of the above coefficients and all individual variables such as age (1 for age ≥75 years; 0 for <75 years), sex (1 for women; 0 for men), BMI (continuous value), and EI (continuous value), was calculated. The EI was calibrated by adding this product to the intercept. The validity of the calibrated EI calculated using this equation was verified by TEE using the DLW method measured in the same group for which the equation was developed (Spearman’s rank correlation coefficient = 0.517).^(12)^ To estimate the ‘true’ group mean of the calibrated EI with an error rate of 0.5% within a 95% confidence interval, a sample size of 682 women and 498 men was required.^(12)^ For both men and women, the correlation coefficient (r) between once-measured calibrated EI ratings and the ‘true’ unmeasured mean calibrated EI was 0.95, indicating that dietary ratings accurately reflect habitual EI.^(12)^ Thus, the sample size and number of daily dietary surveys were sufficient for this study.

As the above equation was developed to reduce systematic errors in EI estimation using the FFQ for older Japanese people, the adaptability of this equation to other populations and dietary survey methods had to be tested. To test the applicability of this calibration formula to other populations, data were extracted from original articles included in a systematic review^(31)^ that compared EI estimated using the self-reported dietary survey method with TEE measured using the DLW method. Of the 31 reports identified in the systematic review,^(31)^ 25 were included in this study, after excluding six reports in which the number of individuals aged ≥75 years was unknown (Supplementary Table 1). We reviewed all 25 articles and extracted data on the number of participants, percentage of women, percentage of participants >75 years of age, mean or median BMI, TEE measured using the DLW method, and EI estimated from each dietary survey.

### Other covariates

American race/ethnicity was categorised as non-Hispanic White or other. In NHANES, height and weight were measured by trained medical technicians using standardised procedures and calibrated equipment.^(32)^ The participants were required to wear a standard MEC examination gown consisting of a disposable shirt, pants, and slippers. Standing height and weight were assessed using a digital scale and wall-mounted digital stadiometer, respectively. In the NNS-J and NHNS-J, participants were barefoot and in light clothing, and their height and weight were assessed using a stadiometer and scale. If these variables could not be measured, or if participants were unable to get to the facility, values were obtained from self-reports or measurements at home. In NHANES, NNS-J, and NHNS-J, height and weight were measured to the nearest 0.1 cm and 0.1 kg, respectively; BMI (kg/m^2^) was calculated as weight (kg) divided by height squared (m^2^).

We considered that assessing both physical activity and EI would enable an understanding of weight changes in terms of energy balance, specifically whether weight gain was due to increased EI or decreased physical activity. The daily step counts of the Japanese participants were measured in November, excluding Sundays and holidays, using a pedometer (ALNESS 200 S AS-200; Yamasa Corporation, Tokyo, Japan). Details of the pedometer survey have been reported previously.^(33)^ Participants were instructed to wear the pedometer on their waist from waking up until going to bed and to remove it only during in-water activities. The participants recorded the number of steps taken and reported whether they wore a pedometer. Our tabulation excluded participants who took fewer than 100 steps per day or more than 50,000 steps per day. These extreme values had already been removed in the original NHNS dataset. Furthermore, we calculated EI per 100 steps to evaluate the association between the amount of physical activity and EI. A previous study has shown that EI per 100 steps is a more accurate predictor of mortality in older Japanese individuals.^(34)^

### Statistical analysis

The analyses were stratified according to sex. Participant characteristics were described using means and standard deviations for continuous variables and numbers and percentages (%) for categorical variables.

The validity of the calibrated EI was confirmed by evaluating the difference between the mean values of the uncalibrated and calibrated EI with the TEE measured by DLW using a paired t-test. The results are presented as mean difference and rate. General linear regression models were used to calculate the annual percentage change (APC) for each variable, including the uncalibrated EI, calibrated EI, BMI, number of steps (Japan only), and EI per 100 steps (Japan only). The *p*-value for the linear trend was calculated by treating exposure as a continuous variable. This study assessed the statistical significance of non-linearity using the Wald test to compare the likelihood ratios of the spline and linear models. A *p*-value less than 0.05 was considered to indicate a statistically significant non-linear relationship between exposure and outcome. Sex-specific analyses were adjusted for age; analyses including all participants were adjusted for both age and sex. In the USA, these adjustments were made within regression models. In Japan, the data were adjusted by sex and age group based on the 2010 survey conducted immediately before the Great East Japan Earthquake.

All statistical analyses used a two-sided significance level of less than 5%, and STATA MP version 18.0 (StataCorp LP, College Station, Texas, USA) was used for all the analyses.

## Results

Table 1 shows a comparison of the uncalibrated and calibrated EI values with the TEE measured using the DLW method. In the analysed 25 studies, the 24HR method was the most frequently used dietary assessment method. The average TEE values measured using the DLW method, uncalibrated EI, and calibrated EI for all studies were 2492, 1988, and 2382 kcal/day, respectively. The calibrated EI was significantly 16.5% higher than the uncalibrated EI (*p* = 0.005). The uncalibrated EI in all surveys was significantly lower than the TEE measured using the DLW method (mean difference: −504 kcal/day; −20.2%, *p* = 0.002). However, the calibrated EI was not significantly different from the TEE (mean difference: −110 kcal/day, −4.1%, *p* = 0.259). The difference between the uncalibrated EI and TEE measured using the DLW method was negatively correlated with BMI (*p* for trend <0.001). However, when the calibrated EI was used, these associations became weaker (Supplementary Table 2).

**Table 1.** Validation of uncalibrated and calibrated energy intake against total energy expenditure measured by doubly labelled water method

	Number of studies^a^	*n*	TEE (kcal/d)			EI (kcal/d)							Difference against TEE (kcal/d or %)				
			DLW			Uncalibrated				Calibrated			Uncalibrated			Calibrated	
			Mean	SD^b^	Range	Mean	SD^b^	Range		Mean	SD^b^	Range	Mean	Rate		Mean	Rate
**Total**																	
**All**	25	7484	2492	460	(1853–3889)	1988	635	(1052–2909)		2382	141	(2104–2765)	−504*	−20.2*		−110	−4.1
24HR	10	3379	2497	447	(2288–3027)	2056	639	(1052–2560)		2405	95	(2142–2524)	−441	−17.7		−92	−3.5
24HR with food photos	2	132	2900	606	(2845–3027)	2870	544	(2783–2909)		2633	128	(2507–2687)	−29	−0.9		−267	−9.1
FFQ	8	2170	2470	440	(2179–3326)	1777	208	(1715–2747)		2331	185	(2167–2765)	−693*	−28.0*		−139	−5.4
Weighed DR	5	135	3103	677	(2626–3889)	2394	395	(2182–2548)		2477	194	(2193–2708)	−709	−22.2		−626	−19.5
Estimated DR	9	1641	2417	382	(1853–2701)	2018	634	(1772–2318)		2377	97	(2104–2417)	−399	−16.3		−40	−1.5
Diet histories	2	27	3212	1056	(2366–3889)	2425	994	(2060–2718)		2381	164	(2252–2484)	−786	−22.5		−831	−22.2
**Women**																	
**All**	23	3590	2196	386	(1734–3630)	1737	517	(1052–2550)		2138	100	(1919–2396)	−459*	−20.7*		−57	−2.3
24HR	9	1635	2060	383	(2079–2591)	1676	556	(1052–2251)		2021	93	(1919–2352)	−427	−19.2		−43	−1.7
24HR with food photos	2	66	2495	553	(2453–2591)	2508	416	(2412–2550)		2330	154	(2179–2396)	13	0.7		−165	−6.4
FFQ	6	931	2150	368	(1955–2277)	1557	316	(1514–1883)		2091	94	(1947–2223)	−593*	−27.3*		−59	−2.6
Weighed DR	4	53	2641	568	(2337–3630)	2028	457	(1912–2182)		2128	54	(2063–2193)	−613	−21.6		−513	−17.7
Estimated DR	9	888	2151	309	(1734–2411)	1760	404	(1432–1924)		2119	76	(1960–2157)	−390*	−17.9*		−32	−1.1
Diet histories	2	17	2923	767	(2294–3630)	1855	399	(1788–1914)		2100	43	(2067–2128)	−1068	−32.7		−823	−24.1
**Men**																	
**All**	22	3328	2792	508	(2125–4115)	2222	694	(1052–3532)		2642	150	(2225–2979)	−570*	−20.5*		−149	−5.1
24HR	9	1511	2611	493	(2390–3462)	2142	718	(1052–2869)		2480	153	(2225–2772)	−493	−17.6		−145	−5.0
24HR with food photos	2	66	3304	656	(3236–2462)	3462	648	(3154–3267)		2935	102	(2834–2979)	−72	−2.0		−369	−11.0
FFQ	6	956	2741	476	(2368–2988)	1948	730	(1889–2380)		2573	103	(2362–2627)	−793*	−28.7*		−168	−6.0
Weighed DR	3	32	3506	904	(3217–4115)	2784	470	(2679–3054)		2684	87	(2605–2780)	−721	−20.2		−821	−22.9
Estimated DR	9	753	2729	437	(2126–3192)	2251	510	(2017–2800)		2651	114	(2410–2763)	−479*	−17.4*		−78	−2.6
Diet histories	2	10	3655	1440	(2580–4115)	3221	1082	(2497–3532)		2813	192	(2623–2894)	−433	−10.9		−842	−20.3

*Note*: TEE, total energy expenditure; DLW, doubly labelled water; EI, energy intake; SD, standard deviation; 24HR, 24 h-diet recalls; FFQ. food frequency questionnaire; Weighed DR, weighed diet records; Estimated DR, weighed diet records. Energy intake conversion factor: 1 kJ = 0.239 kcal. *Indicates statistical significance compared with TEE using a paired t-test (*P* < 0.05).

a In cases where multiple dietary assessment methods were used within a single study, each method was counted separately. Therefore, the total number of studies may have differed from the sum of individual dietary assessment method counts.

b SDs of TEE and EI were calculated based on data available in specific studies.

Table 2 presents the characteristics of the participants included in the national survey data for the USA and Japan. From 2003 to 2018, the proportion of older American adults remained unchanged, whereas that of non-Hispanic adults decreased. In contrast, the proportion of older adults in the Japanese population increased significantly between 1995 and 2019. Both the American and Japanese populations showed no significant changes in the male-to-female ratio.

**Table 2.** Participant characteristics included by data from national survey in the USA and Japan

Survey years	USA													Japan										
	*n*	Age, ≥75 y (*n* [%])^a^		Women (*n* [%])^a^		Height (cm)^b^		Body weight (kg)^b^		BMI (kg/m^2^)^b^		Non-Hispanic, White (*n*, [%])^a^		*n*	Age, ≥75 y (*n* [%])^a^		Women (*n* [%])^a^		Height (cm)^b^		Body weight (kg)^b^		BMI (kg/m^2^)^b^	
1995														9411	669	(7.1)	5228	(55.6)	158.8	(5.6)	57.5	(8.7)	22.7	(3.2)
1996														9326	642	(6.9)	5153	(55.3)	158.9	(5.7)	57.5	(8.8)	22.7	(3.1)
1997														9135	763	(8.4)	5096	(55.8)	158.8	(5.6)	57.6	(8.8)	22.7	(3.2)
1998														9569	747	(7.8)	5278	(55.2)	158.9	(5.7)	58.0	(8.9)	22.9	(3.2)
1999														7997	641	(8.0)	4456	(55.7)	159.1	(5.6)	58.0	(8.9)	22.8	(3.2)
2000														8211	708	(8.6)	4440	(54.1)	159.1	(5.7)	58.1	(9.1)	22.8	(3.3)
2001														8265	768	(9.3)	4640	(56.1)	159.0	(5.8)	58.2	(9.0)	22.9	(3.2)
2002														7647	798	(10.4)	4254	(55.6)	158.9	(5.8)	58.3	(9.1)	23.0	(3.3)
2003	4232	468	(11.1)	2064	(48.8)	168.3	(10.2)	79.6	(19.5)	28.0	(6.1)	2151	(50.8)	7631	881	(11.5)	4262	(55.9)	158.8	(5.8)	58.1	(9.2)	22.9	(3.3)
2004														6186	703	(11.4)	3438	(55.6)	159.7	(5.7)	58.5	(9.1)	22.9	(3.2)
2005	4265	336	(7.9)	2047	(48.0)	168.5	(10.2)	80.9	(21.2)	28.4	(6.7)	2011	(47.2)	5979	773	(12.9)	3315	(55.4)	159.3	(5.7)	58.5	(9.2)	23.0	(3.4)
2006														6530	867	(13.3)	3621	(55.5)	159.4	(5.8)	58.7	(9.1)	23.0	(3.3)
2007	5163	495	(9.6)	2559	(49.6)	167.7	(10.1)	80.8	(20.3)	28.7	(6.5)	2380	(46.1)	6532	859	(13.2)	3592	(55.0)	159.7	(5.7)	58.6	(9.5)	22.9	(3.4)
2008														6756	1034	(15.3)	3734	(55.3)	159.3	(5.7)	58.4	(9.2)	22.9	(3.3)
2009	5532	448	(8.1)	2790	(50.4)	167.7	(10.3)	81.4	(21.1)	28.9	(6.6)	2591	(46.8)	6584	978	(14.9)	3652	(55.5)	159.4	(5.7)	58.5	(9.7)	22.9	(3.5)
2010														6142	894	(14.6)	3399	(55.3)	159.5	(5.7)	58.6	(9.5)	22.9	(3.4)
2011	4654	340	(7.3)	2279	(49.0)	167.8	(10.0)	80.6	(20.7)	28.5	(6.6)	1716	(36.9)	5749	952	(16.6)	3161	(55.0)	159.6	(5.7)	58.8	(9.6)	23.0	(3.5)
2012														21927	3518	(16.0)	12207	(55.7)	159.8	(5.7)	58.6	(9.4)	22.9	(3.4)
2013	4962	351	(7.1)	2511	(50.6)	167.6	(10.1)	80.8	(21.4)	28.7	(6.8)	2139	(43.1)	5991	994	(16.6)	3243	(54.1)	160.0	(5.7)	58.8	(9.7)	22.9	(3.5)
2014														5997	1025	(17.1)	3246	(54.1)	160.0	(5.6)	59.0	(9.7)	22.9	(3.5)
2015	4898	442	(9.0)	2493	(50.9)	166.4	(10.0)	81.8	(21.6)	29.5	(7.0)	1628	(33.2)	5556	948	(17.1)	3068	(55.2)	160.1	(5.8)	59.0	(9.8)	22.9	(3.5)
2016														18903	3530	(18.7)	10406	(55.0)	160.1	(5.3)	59.2	(9.1)	23.0	(3.3)
2017	4664	439	(9.4)	2333	(50.0)	166.8	(10.0)	82.8	(22.4)	29.6	(7.2)	1666	(35.7)	5086	960	(18.9)	2746	(54.0)	160.3	(5.7)	59.8	(9.9)	23.1	(3.5)
2018														5211	943	(18.1)	2805	(53.8)	160.8	(5.8)	59.9	(9.7)	23.1	(3.5)
2019														4308	820	(19.0)	2340	(54.3)	160.4	(5.7)	59.9	(10.1)	23.2	(3.6)

*Note*: BMI, Body mass index. Body mass index was calculated as body weight (kg) divided by height squared (m^2^).

a Categorical values are shown as numbers (percentages).

b Continuous values are shown as mean (standard deviation).

**Figure 2.**
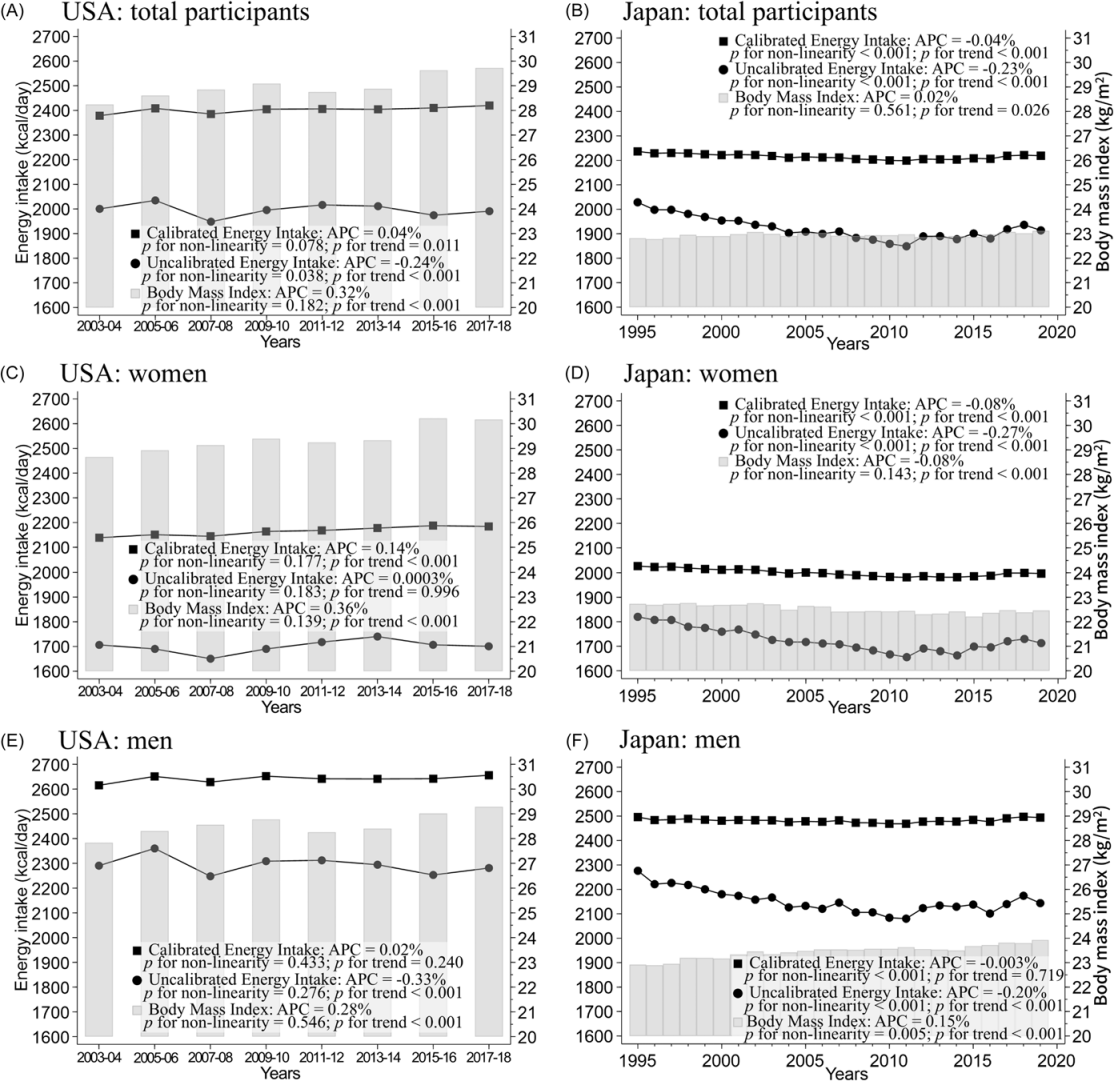
Age-adjusted trends in energy intake with or without biomarker-calibration, and body mass index from 2003 to 2018 in the USA and from 1995 to 2019 in Japan. (A) *n* = 38,370 total participants, (C) *n* = 19,076 women, and (E) *n* = 19,294 men in the USA. (B) *n* = 200,629 total participants, (D) *n* = 110,780 women, (F) *n* = 89,849 in men in Japan. Solid lines represent mean energy intake with (■) or without (●) biomarker-calibration. The histogram shows the distribution of mean body mass index. All values in the age-adjusted model were calculated using regression analysis for the USA and corrected for 2010 age groups according to sex for Japan. The *p*-value of the linear trend was calculated by treating the exposure variable as a continuous variable. Statistical significance of non-linearity was assessed using a Wald test, comparing the likelihood ratio of the spline model with the linear model, and *p*-values of <0.05 were regarded as indicating a statistically significant non-linear relationship between the exposure and outcome. APC, annual percentage change.

Figure 2 shows the trends in age-adjusted EI with and without biomarker calibration and BMI in the USA and Japan. Among Americans, the mean BMI increased during the study period in both sexes (28.7 to 30.2 kg/m^2^ in women, 27.8 to 29.3 kg/m^2^ in men, *p* for trend < 0.001) (Figure 2A, C, E, and Supplementary Table 2). In women, the uncalibrated EI did not change significantly, but the calibrated EI increased by 45 kcal/day (from 2139 to 2184 kcal/day, *p* for trend <0.001) during the 15-year study period (Figure 2C and Supplementary Table 2. In men, the uncalibrated EI decreased by 10 kcal/day (from 2291 to 2281 kcal/day, *p* for trend <0.001), whereas the calibrated EI did not show a significant change. Throughout the study period, the uncalibrated EI was consistently lower than the calibrated EI in both men and women, and the discrepancy rate was greater in women than in men (−20% to −23% in women, −12% to −15% in men) (Supplementary Figure 2A). These relationships were observed in the age-unadjusted data (Supplementary Figures 1A, 1C, 1E, 2B, and Supplementary Table 5).

During the study period, Japanese women experienced a decrease in uncalibrated EI by an average of 107 kcal/day, calibrated EI by an average of 30 kcal/day, and BMI by an average of 0.5 kg/m^2^ (uncalibrated EI: 1820 to 1713 kcal/day; calibrated EI: 2027 to 1997 kcal/day; BMI: 22.7–22.5 kg/m^2^, *p* for trend <0.001). In men, the average BMI increased by 1.0 kg/m^2^, while the average uncalibrated EI decreased by 132 kcal/day (BMI: 22.9–23.9 kg/m^2^, *p* for trend <0.001; uncalibrated EI: 2276–2144 kcal/day, *p* for trend <0.001). However, the average calibrated EI did not change significantly. Without adjustments for age, BMI showed no decrease in women, while the calibrated EI decreased in men (Supplementary Figures 1B, 1D, 1F, and Supplementary Table 6). The uncalibrated EI was lower than the calibrated EI in both men and women, and the discrepancy rates decreased over the study period (*p* for trend <0.001) (Supplementary Figures 2A and 2B).

**Figure 3.**
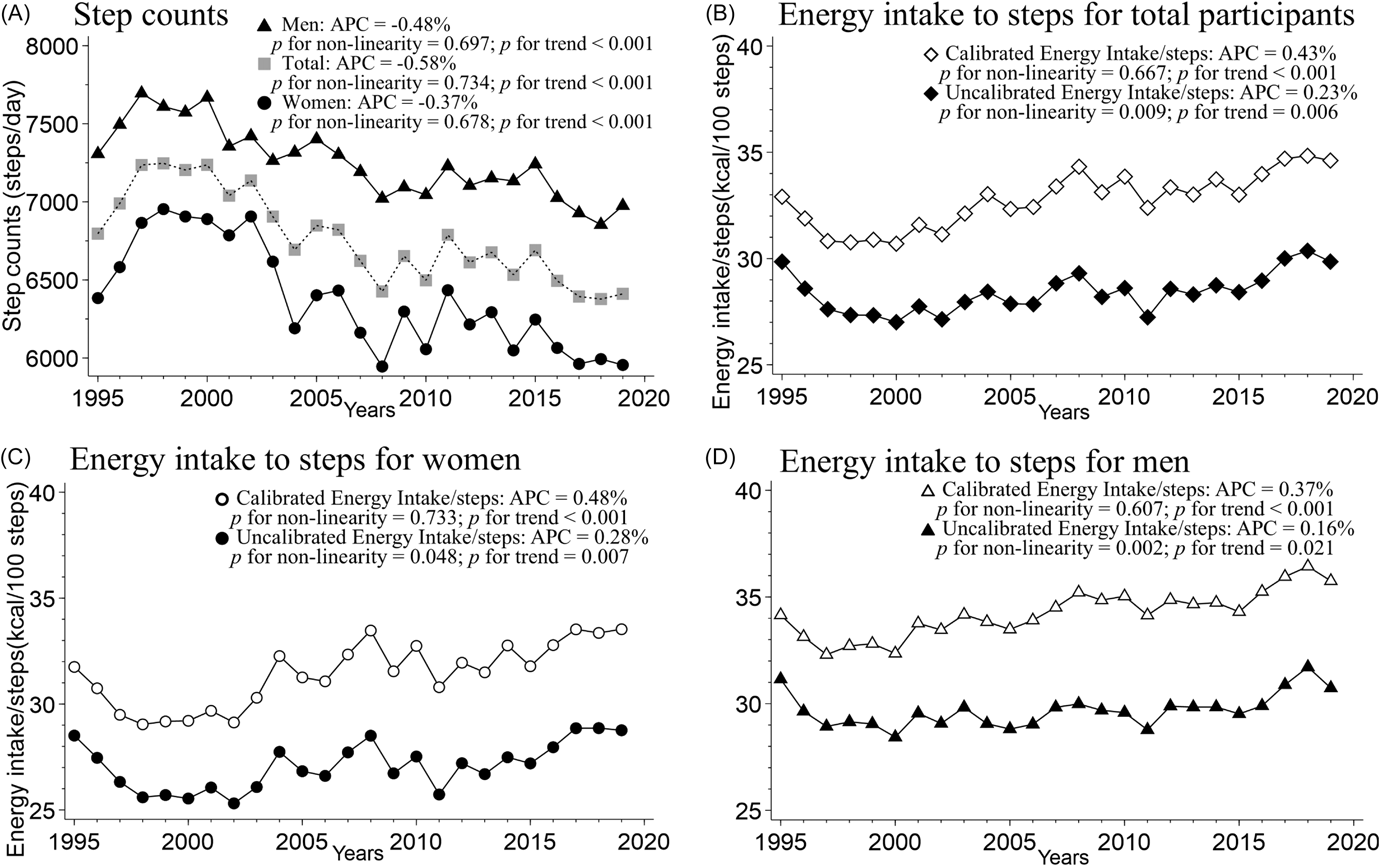
Age-adjusted trends in energy intake to steps, and step counts in Japan. (A) and (B) *n* = 214,463 in total participants, (C) *n* = 97,769 in women, (D) *n* = 116,694 in men. (A) Lines represent mean step counts in men (▲), total participants (■) or women (●). (B), (C), and (D) Solid lines represent mean energy intake with (outlined in white) or without (black paint) biomarker-calibration to steps. All values for age-adjusted model were corrected for the 2010 age category according to sex. The *p*-value of the linear trend was calculated by treating the exposure variable as a continuous variable. Statistical significance of non-linearity was assessed using a Wald test, comparing the likelihood ratio of the spline model with the linear model, and *p*-values of <0.05 were regarded as indicating a statistically significant non-linear relationship between the exposure and outcome. APC, annual percentage change.

Figure 3 shows the trends in age-adjusted step counts and EI for steps in Japan. Between 1995 and 2019, the average number of steps taken by men and women decreased by approximately 1000 steps (Figures 3A–C). The calibrated EI per 100 steps/day increased in both sexes (32–34 kcal/100 steps/day in women and 34–36 kcal/100 steps/day in men; *p* for trend <0.05) (Figure 3D).

## Discussion

This study evaluated the annual changes in BMI, uncalibrated EI, and calibrated EI among American adults (NHANES, 2003–2018) and Japanese adults (NNS-J and NHNS-J, 1995–2019). Over the past several decades, the BMI has increased in both American and Japanese populations. Additionally, the uncalibrated EI decreased in both populations. However, the calibrated EI increased among Americans, and the EI per 100 steps increased among Japanese. For the calibrated EI, no significant changes were observed among men in either Japan or the USA. Among women, calibrated EI decreased in Japanese women but increased in American ones. To the best of our knowledge, this is the first study to evaluate the annual changes in calibrated EI and BMI in Japanese and American adults.

TEE tends to be lower in older individuals, women, those with low levels of physical activity, and those with a lower BMI.^(35)^ In the present study, calibrated EI estimated using the calibration equation also showed lower values in women and individuals with low BMI, consistent with the associations observed for TEE. Similar associations were observed in the population in which the equation was originally developed.^(9)^ Although self-reported EI can only explain a small percentage of the variation in TEE assessed using biomarkers, individual characteristics, particularly BMI, have been shown to explain more biomarker variation than EI.^(9,10,36)^ This may be due to the systematic underreporting of EI in individuals with high BMIs.^(9,10,36)^ Previous studies have reported that the difference between the TEE measured using the DLW method and self-reported EI is reduced after adjusting for variables such as age and BMI.^(10,12,36)^ Our findings indicated that a higher BMI was associated with a greater discrepancy between uncalibrated EI and TEE as measured using the DLW method, implying the difficulty in comparing self-reported EI in populations with widely varying BMIs. However, our results showed that the association between BMI and the discrepancy rate between calibrated EI and TEE measured using the DLW method was weakened. Similar results were obtained in another study examining the validity of a calibration formula.^(12)^ Thus, calibrated EI, which accounts for variables such as sex and BMI, may partially mitigate the impact of systematic errors caused by BMI compared to uncalibrated EI. In addition, previous studies have reported that calibrated EI is associated with mortality risk, whereas uncalibrated EI is not, highlighting the value of the calibration approach.^(16)^

Previous studies have reported an increase in the obesity rate^(1)^ and a decrease in EI^(7,8)^ among American adults and Japanese men in the past few decades. As previously stated, systematic errors in EI tend to be greater in groups with higher BMIs. Therefore, it is unlikely that the EI of Americans and Japanese, whose group-average BMI changed, was accurately assessed. Consistent with prior research, our findings indicate a decrease in uncalibrated EI among American and Japanese men. In American women, both BMI and calibrated EI increased, suggesting that increased EI may contribute to obesity. However, while the BMI of American and Japanese men increased, no differences were observed in the annual changes in calibrated EI. A previous reported that between 2007 and 2016, American adults did not show improved adherence to physical activity guidelines for aerobic exercise, and their sitting time significantly increased.^(37)^ We observed that step count of Japanese men decreased by approximately 840 steps, and their EI per step count increased during the study period. This change can be quantified as a reduction in the energy expenditure of 24 kcal per 100 steps per day. This value was derived from the following equation: ΔEE = 3.0 METs × −8.4 min/h × 65.1 kg, where 3.0 METs is the metabolic equivalent of walking, -8.4 min is the time equivalent of 840 steps (10 min per 1000 steps), and 65.1 kg is the average body weight during the study period. As the calibrated EI showed no significant change during this period, the decrease in EE can be assumed to have exceeded the change in EI. Therefore, in American and Japanese men, a decrease in energy expenditure due to a decrease in physical activity, rather than an increase in EI, may have contributed to the increase in body mass.

In contrast, we showed that Japanese women’s BMI and calibrated EI decreased, and their step count decreased by approximately 1000 steps. Converted to EE using the same method as for Japanese men, this equates to 3.0 METs × −10 min/60 min/h × 52.8 kg = −26 kcal/day. The calibrated EI decreased by 30 kcal/day, suggesting that the decrease in EI was greater than that in EE due to the decrease in physical activity, resulting in a negative energy balance and weight loss. The trends in calibrated EI and BMI in this study are consistent with those in studies examining the energy imbalance gap between Japan and the USA.^(38–40)^ Further studies evaluating EI and energy expenditure in more detail are needed to understand the changes in energy balance that contribute to changes in body mass in the population.

The strength of this study was that it confirmed the validity of the calibrated EI for TEE measured using the DLW method for each dietary survey method. The use of calibrated EI measurements is important for reducing systematic errors in the EI calculated from self-reported dietary assessments and for verifying the actual status of EI and its temporal trends. However, this study had some limitations. First, we calculated the calibrated EI using the same coefficients for Japanese and American participants. Our calibration equation was originally developed for a Japanese population, in which severe obesity is relatively rare. For example, if the relationship between BMI and EI underreporting is exponential, the accuracy of the equation may be limited in populations with a higher prevalence of severe obesity, such as that in the USA. As the impact of systematic errors in each coefficient may differ between Japan and the USA, direct comparisons of the EI between the two countries based on these results are not possible. Second, this was a repeated cross-sectional study with different respondents for each survey year. Therefore, our data cannot indicate within-individual changes in EI or BMI but are limited to population-level changes. Consequently, this study could not establish a causal relationship between changes in BMI and EI. Third, the response rates for the NHANES, NNS-J, and NHNS-J were approximately 45 to 70%, and sample weights were not available for the stratified analysis of the NHANES. These sampling biases may have affected our results. In addition, the relationship between BMI and EI changed after adjustment for age in the Japanese population, implying that these variables may have been influenced by other factors that change with ageing in the Japanese population. Fourth, the food standard tables of the ingredients used to calculate EI differed between surveys because they were revised. EI was estimated from data of a dietary survey covering 1 day for Japanese participants and at least one day for Americans, which may not reflect the habitual dietary intake of the participants. However, because the same protocol has been used for many years, this factor may not have a significant impact when assessing relative changes within the same population. In addition, although multi-day dietary assessments improve the accuracy of the individual ranking of dietary intake, in studies such as the current one that evaluate the population mean, a single-day assessment can provide valid estimates when the sample size is sufficiently large; thus, the use of one-day dietary data is unlikely to have substantially affected our results. These limitations may limit the generalisability of our results and indicate the need for studies that longitudinally assess EI and EE in the same individuals in an objective manner rather than through self-reported dietary assessments, such as the DR and 24HR.^(41)^

The calibration method is considered a more effective approach for the precise assessment of EI in large populations where obtaining accurate dietary biomarkers is difficult, and for trend analyses, such as in the present study, where data cannot be re-collected. However, as previously noted, this approach has limitations when applied to populations with characteristics that differ from those used to develop the calibration equation. Therefore, creating calibration equations from populations that include, for example, smaller-bodied East Asian individuals and larger-bodied Western individuals could enable the use of a single regression equation to adjust EI even in populations with substantially different attributes. This strategy is not limited to discussions of aggregated trend analyses, such as those in our study, but could be applied to dietary survey data from large-scale cohort studies conducted worldwide, thereby contributing to a better understanding of the relationship between diet and disease.

## Conclusion

Our results suggest that the decline in self-reported EI over the past several decades in the USA and Japan has been influenced by a systematic bias in dietary surveys. These findings suggest that a system to monitor national EI and physical activity through accurate assessments, rather than relying on self-reports, is necessary to promote adequate EI to maintain an appropriate body mass.

## Supporting information

Inoue et al. supplementary material Inoue et al. supplementary material

## Data Availability

The National Health and Nutrition Examination Survey data is publicly available and freely available on the NCHD website at [https://wwwn.cdc.gov/nchs/nhanes/Default.aspx]. Annual reports and summary tables of the National Nutrition Survey and the National Health and Nutrition Survey, Japan are published on the official website of the survey by the Ministry of Health, Labour and Welfare. Results are published each year in the form of electronically available aggregated data [http://www.mhlw.go.jp/bunya/kenkou/kenkou_eiyou_chousa.html]. Moreover, individual-level data from the National Health and Nutrition Survey, Japan are electronically available for scientific research if approved by the Ministry of Health, Labour and Welfare through official application procedures under Article 33 of the Statistics Act; however, currently, these data are only available in Japanese.

## Supplementary material

The supplementary material for this article can be found at https://doi.org/10.1017/jns.2025.10069
