# Supplementary material for: Trends in body mass index and energy intake with and without biomarker calibration in the USA and Japanese National Nutrition Surveys
Source: J Nutr Sci. 2026 Jan 21;15:e11. doi: 10.1017/jns.2025.10069 (PMC12823207; doi:10.1017/jns.2025.10069)
Supplement: Inoue et al. supplementary material [file S2048679025100694sup001.docx]

**Supplemental Data**

“Trends in body mass index and energy intake with and without biomarker calibration in the USA and Japanese National Nutrition Surveys”

**SUPPLEMENTARY TABLES**

**Supplementary Table 1.** Characteristics of included studies for meta-analysis

**Supplementary Table 2.** Validation of uncalibrated and calibrated energy intake against total energy expenditure measured by doubly labelled water method by weight status

**Supplementary Table 3.** Age-adjusted energy intake and body mass index for USA

**Supplementary Table 4.** Age-adjusted energy intake and body mass index for Japan

**Supplementary Table 5.** Age-unadjusted energy intake and body mass index for USA

**Supplementary Table 6.** Age-unadjusted energy intake and body mass index for Japan

**SUPPLEMENTAL FIGURES**

**Supplementary Figure 1.** Age-unadjusted trends in energy intake with or without biomarker-calibration, and body mass index from 2003 to 2018 in USA and from 1995 to 2019 in Japan

**Supplementary Figure 2.** Trends in under estimate rate of energy intake from 2003 to 2018 in USA and from 1995 to 2019 in Japan

**Supplementary Table 1. Characteristics of included studies for meta-analysis**

| Study | *n* | Women  (%) | Age, >75 years (%) | BMI (kg/m^2^) | | | TEE (kcal/d) | | | EI (kcal/d) | | | CEI (kcal/d) | | | Difference against DLW | | | | | | | | | | | |
| --- | --- | --- | --- | --- | --- | --- | --- | --- | --- | --- | --- | --- | --- | --- | --- | --- | --- | --- | --- | --- | --- | --- | --- | --- | --- | --- | --- |
|  |  |  |  |  |  |  |  |  |  |  |  |  |  |  |  | Uncalibrated (mean[kcal/d], [%]) | | | | | | Calibrated (mean[kcal/d], [%]) | | | | | |
|  |  |  |  | Total | Women | Men | Total | Women | Men | Total | Women | Men | Total | Women | Men | Total | | Women | | men | | Total | | Women | | men | |
| **24HR** | | | | | | | | | | | | | | | | | | | | | | | | | | | |
| Foster 2019 | 98 | 51 | 0 | 26.6 | 26.6 | 26.6 | 2870 | 2498 | 3234 | 2124 | 1914 | 2342 | 2393 | 2169 | 2627 | -746 | -26.0 | -584 | -23.4 | -891 | -27.6 | -477 | -16.6 | -328 | zz | -607 | -18.8 |
| Orcholski 2015 (USA) | 63 | 47.6 | 0 | 30.9 | 34.0 | 28.0 | 2743 | 2342 | 3107 | 2164 | 1888 | 2414 | 2524 | 2352 | 2680 | -579 | -21.1 | -454 | -19.4 | -693 | -22.3 | -219 | -8.0 | 10 | 0.4 | -427 | -13.8 |
| Orcholski 2015 (Seychells) | 72 | 51.4 | 0 | 27.1 | 29.0 | 25.0 | 2548 | 2223 | 2892 | 1848 | 1697 | 2008 | 2337 | 2178 | 2506 | -700 | -27.5 | -526 | -23.7 | -884 | -30.6 | -211 | -8.3 | -44 | -2.0 | -386 | -13.4 |
| Orcholski 2015 (Jamaica) | 63 | 54 | 0 | 25.7 | 28.0 | 23.0 | 2288 | 2079 | 2533 | 1838 | 1673 | 2032 | 2291 | 2147 | 2460 | -450 | -19.7 | -406 | -19.5 | -502 | -19.8 | 3 | 0.1 | 68 | 3.3 | -73 | -2.9 |
| Orcholski 2015 (South Africa) | 59 | 66.1 | 0 | 28.9 | 32.0 | 23.0 | 2343 | 2318 | 2390 | 1052 | 1052 | 1052 | 2142 | 2100 | 2225 | -1291 | -55.1 | -1267 | -54.6 | -1338 | -56.0 | -200 | -8.5 | -218 | -9.4 | -165 | -6.9 |
| Orcholski 2015 | 67 | 53.7 | 0 | 24.1 | 26.0 | 22.0 | 2622 | 2390 | 2892 | 1982 | 1816 | 2175 | 2287 | 2130 | 2469 | -640 | -24.4 | -574 | -24.0 | -717 | -24.8 | -335 | -12.8 | -260 | -10.9 | -423 | -14.6 |
| Gemming 2015 | 40 | 50.0 | 0 | 24.7 | 22.3 | 27.1 | 3027 | 2591 | 3462 | 2560 | 2251 | 2869 | 2453 | 2140 | 2766 | -466 | -15.4 | -340 | -13.1 | -593 | -17.1 | -573 | -18.9 | -451 | -17.4 | -696 | -20.1 |
| Lopes 2016 | 83 | 60.2 | 0 | 31.3 | 21.7 | 35.0 | 2539 | 2411 | 2733 | 1656 | 1395 | 2052 | 2369 | 1919 | 2772 | -883 | -34.8 | -1016 | -42.1 | -681 | -24.9 | -170 | -6.7 | -492 | -20.4 | 39 | 1.4 |
| Pfrimer 2015 | 41 | 51.2 | 0 | 27.5 | 29.0 | 26.0 | 2419 | 2220 | 2627 | 1927 | 1616 | 2253 | 2369 | 2159 | 2590 | -492 | -20.3 | -604 | -27.2 | -374 | -14.2 | -49 | -2.0 | -61 | -2.7 | -37 | -1.4 |
| Moshfegh 2008 | 524 | 50.0 | 0 | 26.6 | 26.3 | 27.0 | 2534 | 2196 | 2873 | 2244 | 1927 | 2561 | 2426 | 2164 | 2689 | -290 | -11.5 | -269 | -12.3 | -311 | -10.8 | -108 | -4.3 | -33 | -1.5 | -183 | -6.4 |
| Subar 2003 | 484 | 46.1 | 0 | 28.0 | 27.4 | 28.4 | 2588 | 2277 | 2849 | 2255 | 1999 | 2474 | 2477 | 2211 | 2705 | -333 | -12.9 | -278 | -12.2 | -375 | -13.2 | -110 | -4.3 | -66 | -2.9 | -144 | -5.1 |
| Mossavar-Rahmani 2015 | 477 | 60.4 | 0 | 29.6 | 29.6 | 29.6 | 2388 | 2170 | 2721 | 1796 | 1579 | 2127 | 2358 | 2165 | 2652 | -592 | -24.8 | -591 | -27.2 | -594 | -21.8 | -30 | -1.3 | -5 | -0.2 | -69 | -2.5 |
| Park 2018 | 1075 | 50.7 | 0 | 28.8 | 27.9 | 29.7 | 2438 | 2136 | 2748 | 2044 | 1807 | 2274 | 2431 | 2177 | 2689 | -394 | -16.2 | -329 | -15.4 | -474 | -17.2 | -7 | -0.3 | 41 | 1.9 | -59 | -2.1 |
| Arab 2011 | 233 | 67.8 | 0 | (25.0) | (25.0) | (25.0) | 2445 |  |  | 2222 |  |  | 2316 |  |  | -223 | -9.1 |  |  |  |  | -129 | -5.3 |  |  |  |  |
| **24HR with food photos** | | | | | | | | | | | | | | | | | | | | | | | | | | | |
| Ptomey 2015 | 92 | 50 | 0 | 30.6 | 29.5 | 31.7 | 2845 | 2453 | 3236 | 2909 | 2550 | 3267 | 2687 | 2396 | 2979 | 64 | 2.2 | 97 | 4.0 | 31 | 1.0 | -157 | -5.5 | -57 | -2.3 | -257 | -7.9 |
| Gemming 2015 | 40 | 50 | 0 | 24.7 | 22.3 | 27.1 | 3027 | 2591 | 3462 | 2783 | 2412 | 3154 | 2507 | 2179 | 2834 | -244 | -8.1 | -179 | -6.9 | -308 | -8.9 | -520 | -17.2 | -412 | -15.9 | -628 | -18.1 |
| **FFQ** |  |  |  |  |  |  |  |  |  |  |  |  |  |  |  |  |  |  |  |  |  |  |  |  |  |  |  |
| Okubo 2008 | 140 | 52.1 | 0 | 22.4 | 21.6 | 23.3 | 2258 | 1984 | 2557 | 1953 | 1804 | 2115 | 2241 | 2015 | 2488 | -305 | -13.5 | -179 | -9.0 | -442 | -17.3 | -17 | -0.7 | 31 | 1.6 | -69 | -2.7 |
| Pfrimer 2015 | 41 | 51.2 | 0 | 27.5 | 29.0 | 26.0 | 2419 | 2220 | 2627 | 2121 | 1883 | 2380 | 2416 | 2223 | 2620 | -298 | -12.3 | -337 | -15.2 | -247 | -9.4 | -3 | -0.1 | 3 | 0.1 | -7 | -0.3 |
| Nybacka 2016 | 40 | 50 | 0 | 26.5 | 25.7 | 27.3 | 2569 | 2151 | 2988 | 2009 | 1769 | 2271 | 2367 | 2111 | 2627 | -560 | -21.8 | -382 | -17.8 | -717 | -24.0 | -203 | -7.9 | -40 | -1.8 | -360 | -12.1 |
| Watanabe 2019 | 109 | 45.9 | 35.8 | 22.8 | 23.0 | 22.7 | 2179 | 1955 | 2368 | 1756 | 1619 | 1905 | 2167 | 1947 | 2362 | -423 | -19.4 | -336 | -17.2 | -463 | -19.6 | -11 | -0.5 | -8 | -0.4 | -6 | -0.2 |
| Subar 2003 | 482 | 46.1 | 0 | 28.0 | 27.4 | 28.4 | 2588 | 2277 | 2849 | 1716 | 1514 | 1889 | 2348 | 2094 | 2564 | -872 | -33.7 | -763 | -33.5 | -960 | -33.7 | -240 | -9.3 | -183 | -8.0 | -285 | -10.0 |
| Svendsen 2006 | 50 | 54 | 0 | 35.7 | 36.6 | 34.6 | 3326 |  |  | 2747 |  |  | 2765 |  |  | -579 | -17.4 |  |  |  |  | -561 | -16.9 |  |  |  |  |
| Park 2018 | 1075 | 50.7 | 0 | 28.8 | 27.9 | 29.7 | 2438 | 2136 | 2748 | 1715 | 1516 | 1932 | 2352 | 2107 | 2607 | -723 | -29.7 | -620 | -29.0 | -816 | -29.7 | -86 | -3.5 | -29 | -1.3 | -141 | -5.1 |
| Arab 2011 | 233 | 67.8 | 0 | (25.0) | (25.0) | (25.0) | 2445 |  |  | 1783 |  |  | 2211 |  |  | -662 | -27.1 |  |  |  |  | -234 | -9.6 |  |  |  |  |
| **Weighed DR** | | | | | | | | | | | | | | | | | | | | | | | | | | | |
| Livingstone 1990 | 31 | 48.4 | 0 | 25.1 | 24.3 | 25.8 | 2904 | 2373 | 3401 | 2308 | 1912 | 2679 | 2408 | 2110 | 2687 | -596 | -20.5 | -461 | -19.4 | -722 | -21.2 | -496 | -17.1 | -263 | -11.1 | -714 | -21.0 |
| Barnard 2002 | 15 | 53.3 | 0 | 24.9 | 23.8 | 25.9 | 3889 | 3630 | 4115 | 2548 | 1970 | 3054 | 2443 | 2111 | 2780 | -1341 | -34.5 | -1660 | -45.7 | -1061 | -25.8 | -1446 | -37.2 | -1519 | -41.8 | -1336 | -32.5 |
| Warwick 1996 | 21 | 57.1 | 0 | 21.8 | 21.8 | 21.8 | 2714 | 2337 | 3217 | 2314 | 1980 | 2760 | 2295 | 2063 | 2605 | -400 | -14.7 | -357 | -15.3 | -457 | -14.2 | -419 | -15.4 | -274 | -11.7 | -612 | -19.0 |
| Black 1997 | 18 | 100 | 0 | 25.0 | 25.0 | 25.4 | 2626 | 2626 |  | 2182 | 2182 |  | 2193 | 2193 |  | -444 | -16.9 | -444 | -16.9 |  |  | -433 | -16.5 | -433 | -16.5 |  |  |
| Svendsen 2006 | 50 | 54 | 0 | 35.7 | 36.6 | 34.6 | 3326 |  |  | 2512 |  |  | 2708 |  |  | -814 | -24.5 |  |  |  |  | -618 | -18.6 |  |  |  |  |
| **Estimated DR** | | | | | | | | | | | | | | | | | | | | | | | | | | | |
| Goran 1992 | 13 | 46.2 | 7.7 | 24.6 | 24.0 | 25.2 | 2406 | 2092 | 2675 | 1913 | 1432 | 2326 | 2296 | 1973 | 2573 | -493 | -20.5 | -660 | -31.5 | -349 | -13.0 | -110 | -4.6 | -119 | -5.7 | -102 | -3.8 |
| Koebnick 2005 | 29 | 55.2 | 0 | 23.4 | 23.4 | 23.4 | 2701 | 2306 | 3192 | 2318 | 1918 | 2800 | 2343 | 2088 | 2655 | -383 | -14.2 | -389 | -16.9 | -392 | -12.3 | -357 | -13.2 | -218 | -9.5 | -538 | -16.8 |
| Redman 2014 | 217 | 69.6 | 0 | 25.2 | 24.9 | 25.8 | 2444 | 2267 | 2851 | 2318 | 1924 | 2503 | 2338 | 2128 | 2645 | -126 | -5.2 | -343 | -15.1 | -348 | -12.2 | -107 | -4.4 | -139 | -6.1 | -206 | -7.2 |
| Seale 1997 | 19 | 57.9 | 0 | 23.9 | 22.6 | 25.7 | 2623 | 2287 | 3086 | 1992 | 1883 | 2141 | 2268 | 2060 | 2556 | -631 | -24.1 | -404 | -17.7 | -944 | -30.6 | -355 | -13.5 | -228 | -10.0 | -530 | -17.2 |
| Lopes 2016 | 83 | 60.2 | 0 | 31.3 | 21.7 | 35.0 | 2539 | 2411 | 2733 | 1772 | 1611 | 2017 | 2397 | 1971 | 2763 | -767 | -30.2 | -800 | -33.2 | -716 | -26.2 | -142 | -5.6 | -440 | -18.2 | 30 | 1.1 |
| Nybacka 2016 | 40 | 50 | 0 | 26.5 | 25.7 | 27.3 | 2569 | 2151 | 2988 | 1984 | 1745 | 2223 | 2361 | 2106 | 2616 | -586 | -22.8 | -406 | -18.9 | -765 | -25.6 | -209 | -8.1 | -45 | -2.1 | -372 | -12.4 |
| Watanabe 2019 | 109 | 45.9 | 35.8 | 22.8 | 23.0 | 22.7 | 2179 | 1955 | 2368 | 1972 | 1815 | 2105 | 2219 | 1994 | 2410 | -207 | -9.5 | -140 | -7.2 | -263 | -11.1 | 41 | 1.9 | 39 | 2.0 | 42 | 1.8 |
| Takae 2019 | 56 | 69.6 | 33.9 | 23.0 | 22.6 | 23.9 | 1853 | 1734 | 2126 | 1814 | 1704 | 2066 | 2104 | 1960 | 2435 | -39 | -2.1 | -30 | -1.7 | -60 | -2.8 | 251 | 13.6 | 226 | 13.0 | 309 | 14.5 |
| Park 2018 | 1075 | 50.7 | 0 | 28.8 | 27.9 | 29.7 | 2438 | 2136 | 2748 | 1986 | 1725 | 2244 | 2417 | 2157 | 2682 | -452 | -18.5 | -411 | -19.2 | -504 | -18.3 | -21 | -0.8 | 21 | 1.0 | -66 | -2.4 |
| **Diet histories** | | | | | | | | | | | | | | | | | | | | | | | | | | | |
| Rothenberg 1998 | 12 | 75 | 0 | 25.0 | 25.0 | 25.0 | 2366 | 2294 | 2580 | 2060 | 1914 | 2497 | 2252 | 2128 | 2623 | -306 | -12.9 | -380 | -16.6 | -83 | -3.2 | -114 | -4.8 | -166 | -7.2 | 43 | 1.7 |
| Barnard 2002 | 15 | 53.3 | 0 | 24.9 | 23.8 | 25.9 | 3889 | 3630 | 4115 | 2718 | 1788 | 3532 | 2484 | 2067 | 2894 | -1171 | -30.1 | -1842 | -50.7 | -583 | -14.2 | -1405 | -36.1 | -1563 | -43.1 | -1221 | -29.7 |

TEE, total energy expenditure; DLW, doubly labelled water; EI, energy intake; SD, standard deviation; 24HR, 24 h-diet recalls; FFQ. food frequency questionnaire; Weighed DR, weighed diet records; Estimated DR, weighed diet records. Energy intake conversion factor: 1 kJ = 0.239 kcal.

BMI values in studies where only median is available are represented in parentheses.

**Supplementary Table 2.** Validation of uncalibrated and calibrated energy intake against total energy expenditure measured by doubly labelled water method by weight status

|  | Number  of  studies^a^ | *n* | BMI (kg/m^2^) | Age, 75 years (%) | TEE (kcal/d) | | |  | EI (kcal/d) | | | | | | |  | Difference against TEE  (kcal/d or %) | | | | |
| --- | --- | --- | --- | --- | --- | --- | --- | --- | --- | --- | --- | --- | --- | --- | --- | --- | --- | --- | --- | --- | --- |
|  |  |  |  |  | DLW | | |  | Uncalibrated | | |  | Calibrated | | |  | Uncalibrated | |  | Calibrated | |
|  |  |  |  |  | Mean | SD^b^ | Range |  | Mean | SD^b^ | Range |  | Mean | SD | Range |  | Mean | Rate |  | Mean | Rate |
| **Total** |  |  |  |  |  |  |  |  |  |  |  |  |  |  |  |  |  |  |  |  |  |
| Normal weight | 13 | 673 | 23.3 | 14.6 | 2445 | 494 | (1853-3889) |  | 2059 | 478 | (1756-2783) |  | 2265 | 125 | (2104-2507) |  | -387 | -15.1 |  | -180 | -5.4 |
| Overweight | 21 | 6390 | 28.0 | 0 | 2475 | 437 | (2288-2904) |  | 1963 | 677 | (1052-2318) |  | 2383 | 85 | (2142-2477) |  | -512 | -20.7 |  | -92 | -3.6 |
| Obese | 6 | 421 | 32.1 | 0 | 2823 | 539 | (2539-3326) |  | 2260 | 577 | (1656-2909) |  | 2554 | 169 | (2765-2369) |  | -563 | -20.5 |  | -269 | -9.0 |
| *p* for trend |  |  |  |  |  |  |  |  |  |  |  |  |  |  |  |  | *p* < 0.001 | |  | *p*  < 0.01 | |
| **Women** |  |  |  |  |  |  |  |  |  |  |  |  |  |  |  |  |  |  |  |  |  |
| Normal weight | 16 | 579 | 23.1 | 8.5 | 2230 | 355 | (1734-3630) |  | 1807 | 383 | (1395-2412) |  | 2039 | 78 | (1919-2179) |  | -423 | -17.8 |  | -191 | -7.1 |
| Overweight | 18 | 2942 | 27.8 | 0 | 2186 | 398 | (2079-2626) |  | 1730 | 568 | (1514-2550) |  | 2156 | 72 | (2094-2396) |  | -455 | -20.9 |  | -29 | -1.2 |
| Obese | 2 | 69 | 32.9 | 0 | 2329 | 539 | (2318-2342) |  | 1415 | 476 | (1052-1888) |  | 2210 | 178 | (2100-2352) |  | -913 | -39.3 |  | -119 | -5.1 |
| *p* for trend |  |  |  |  |  |  |  |  |  |  |  |  |  |  |  |  | *p* < 0.001 | |  | *p < 0.001* | |
| **Men** |  |  |  |  |  |  |  |  |  |  |  |  |  |  |  |  |  |  |  |  |  |
| Normal weight | 9 | 304 | 22.9 | 15.8 | 2527 | 440 | (2126-3217) |  | 2046 | 503 | (1052-2800) |  | 2434 | 126 | (2225-2655) |  | -481 | -19.0 |  | -93 | -3.0 |
| Overweight | 23 | 2912 | 28.8 | 0.0 | 2814 | 519 | (2627-4115) |  | 2228 | 739 | (1889-3532) |  | 2656 | 91 | (2556-2894) |  | -586 | -20.8 |  | -158 | -5.4 |
| Obese | 3 | 112 | 33.6 | 0 | 2940 | 550 | (2733-3236) |  | 2541 | 590 | (2017-3267) |  | 2854 | 122 | (2763-2979) |  | -399 | -14.7 |  | -85 | -2.5 |
| *p* for trend |  |  |  |  |  |  |  |  |  |  |  |  |  |  |  |  | *p* < 0.001 | |  | *p* < 0.001 | |

TEE, total energy expenditure; DLW, doubly labelled water; EI, energy intake; SD, standard deviation; BMI, Body mass index. Energy intake conversion factor: 1 kJ = 0.239 kcal. Body mass index was calculated as body weight (kg) divided by height squared (m^2^). Weight status was classified according to the following BMI categories: normal weight < 25.0 kg/m^2^; overweight 25.0-29.9 kg/m^2^; obese ≥ 30.0 kg/m^2^. *P* value < 0.05 was considered to indicate a significant linear relationship between the differences TEE and EI and BMI based on regression analysis weighted by number of participants (*P* < 0.05).

^a^ In cases where multiple dietary assessment methods were used within a single study, each method is counted separately. Therefore, the total number of studies may differ from the sum of individual dietary assessment method counts.

^b^ SDs of TEE and EI were calculated based on data available in specific studies.

**Supplementary Table 3. Age-adjusted energy intake and body mass index for USA**

| Survey  years | *n* |  | Uncalibrated energy intake (kcal/d) | | | | | |  | Calibrated energy intake (kcal/d) | | | | | |  | Body mass index (kg/m^2^) | | | | | |
| --- | --- | --- | --- | --- | --- | --- | --- | --- | --- | --- | --- | --- | --- | --- | --- | --- | --- | --- | --- | --- | --- | --- |
|  |  |  | Total | | Women | | Men | |  | Total | | Women | | Men | |  | Total | | Women | | Men | |
| 2003 | 4232 |  | 2000 | (845) | 1706 | (635) | 2291 | (924) |  | 2379 | (357) | 2139 | (247) | 2615 | (283) |  | 28.2 | (5.9) | 28.7 | (6.5) | 27.8 | (5.2) |
| 2004 |  |  |  |  |  |  |  |  |  |  |  |  |  |  |  |  |  |  |  |  |  |  |
| 2005 | 4265 |  | 2035 | (835) | 1690 | (600) | 2360 | (894) |  | 2408 | (363) | 2151 | (250) | 2651 | (275) |  | 28.6 | (6.5) | 28.9 | (7.1) | 28.3 | (5.9) |
| 2006 |  |  |  |  |  |  |  |  |  |  |  |  |  |  |  |  |  |  |  |  |  |  |
| 2007 | 5163 |  | 1948 | (856) | 1651 | (626) | 2248 | (946) |  | 2386 | (360) | 2145 | (245) | 2628 | (288) |  | 28.8 | (6.3) | 29.1 | (6.8) | 28.6 | (5.7) |
| 2008 |  |  |  |  |  |  |  |  |  |  |  |  |  |  |  |  |  |  |  |  |  |  |
| 2009 | 5532 |  | 1996 | (827) | 1690 | (596) | 2309 | (910) |  | 2405 | (358) | 2164 | (249) | 2652 | (276) |  | 29.1 | (6.4) | 29.4 | (7.0) | 28.7 | (5.8) |
| 2010 |  |  |  |  |  |  |  |  |  |  |  |  |  |  |  |  |  |  |  |  |  |  |
| 2011 | 4654 |  | 2016 | (817) | 1718 | (627) | 2312 | (874) |  | 2406 | (353) | 2168 | (252) | 2642 | (272) |  | 28.7 | (6.4) | 29.2 | (7.1) | 28.3 | (5.7) |
| 2012 |  |  |  |  |  |  |  |  |  |  |  |  |  |  |  |  |  |  |  |  |  |  |
| 2013 | 4962 |  | 2011 | (837) | 1740 | (662) | 2295 | (904) |  | 2404 | (353) | 2178 | (257) | 2641 | (276) |  | 28.9 | (6.6) | 29.3 | (7.2) | 28.4 | (5.8) |
| 2014 |  |  |  |  |  |  |  |  |  |  |  |  |  |  |  |  |  |  |  |  |  |  |
| 2015 | 4898 |  | 1975 | (797) | 1707 | (633) | 2253 | (853) |  | 2410 | (352) | 2188 | (262) | 2642 | (275) |  | 29.6 | (6.8) | 30.2 | (7.5) | 29.0 | (6.0) |
| 2016 |  |  |  |  |  |  |  |  |  |  |  |  |  |  |  |  |  |  |  |  |  |  |
| 2017 | 4664 |  | 1991 | (849) | 1701 | (636) | 2281 | (933) |  | 2420 | (364) | 2184 | (265) | 2656 | (289) |  | 29.7 | (6.9) | 30.2 | (7.6) | 29.3 | (6.1) |
| 2018 |  |  |  |  |  |  |  |  |  |  |  |  |  |  |  |  |  |  |  |  |  |  |

All values are shown as mean (standard deviation).

**Supplementary Table 4. Age-adjusted energy intake and body mass index for Japan**

| Survey  years | *n* |  | Uncalibrated energy intake (kcal/d) | | | | | |  | Calibrated energy intake (kcal/d) | | | | | |  | Body mass index (kg/m^2^) | | | | | |
| --- | --- | --- | --- | --- | --- | --- | --- | --- | --- | --- | --- | --- | --- | --- | --- | --- | --- | --- | --- | --- | --- | --- |
|  |  |  | Total | | Women | | Men | |  | Total | | Women | | Men | |  | Total | | Women | | Men | |
| 1995 | 9411 |  | 2029 | 609 | 1820 | 497 | 2276 | 638 |  | 2236 | N/A | 2027 | N/A | 2496 | N/A |  | 22.8 | 3.2 | 22.7 | 3.4 | 22.9 | 3.0 |
| 1996 | 9326 |  | 1998 | 601 | 1807 | 504 | 2221 | 632 |  | 2229 | N/A | 2023 | N/A | 2483 | N/A |  | 22.8 | 3.2 | 22.7 | 3.3 | 22.9 | 3.0 |
| 1997 | 9135 |  | 1998 | 582 | 1807 | 490 | 2226 | 605 |  | 2231 | N/A | 2025 | N/A | 2486 | N/A |  | 22.8 | 3.2 | 22.7 | 3.4 | 23.0 | 3.0 |
| 1998 | 9569 |  | 1981 | 582 | 1780 | 483 | 2218 | 602 |  | 2229 | N/A | 2019 | N/A | 2489 | N/A |  | 23.0 | 3.2 | 22.8 | 3.4 | 23.2 | 3.0 |
| 1999 | 7997 |  | 1969 | 578 | 1775 | 490 | 2200 | 595 |  | 2225 | N/A | 2015 | N/A | 2485 | N/A |  | 22.9 | 3.2 | 22.7 | 3.4 | 23.2 | 3.0 |
| 2000 | 8211 |  | 1954 | 580 | 1760 | 492 | 2180 | 596 |  | 2222 | N/A | 2012 | N/A | 2481 | N/A |  | 22.9 | 3.3 | 22.7 | 3.4 | 23.2 | 3.1 |
| 2001 | 8265 |  | 1953 | 598 | 1768 | 497 | 2174 | 636 |  | 2224 | N/A | 2014 | N/A | 2484 | N/A |  | 23.0 | 3.3 | 22.7 | 3.4 | 23.3 | 3.1 |
| 2002 | 7647 |  | 1937 | 561 | 1748 | 468 | 2158 | 583 |  | 2222 | N/A | 2012 | N/A | 2483 | N/A |  | 23.1 | 3.3 | 22.8 | 3.5 | 23.5 | 3.2 |
| 2003 | 7631 |  | 1930 | 589 | 1726 | 482 | 2167 | 619 |  | 2218 | N/A | 2005 | N/A | 2482 | N/A |  | 23.0 | 3.3 | 22.7 | 3.5 | 23.3 | 3.2 |
| 2004 | 6186 |  | 1903 | 548 | 1717 | 455 | 2126 | 573 |  | 2211 | N/A | 1997 | N/A | 2475 | N/A |  | 22.9 | 3.3 | 22.5 | 3.3 | 23.4 | 3.2 |
| 2005 | 5979 |  | 1909 | 585 | 1718 | 485 | 2133 | 607 |  | 2214 | N/A | 2001 | N/A | 2478 | N/A |  | 23.0 | 3.4 | 22.6 | 3.5 | 23.5 | 3.2 |
| 2006 | 6530 |  | 1900 | 571 | 1711 | 466 | 2120 | 599 |  | 2212 | N/A | 1999 | N/A | 2476 | N/A |  | 23.0 | 3.3 | 22.6 | 3.4 | 23.5 | 3.1 |
| 2007 | 6532 |  | 1909 | 581 | 1708 | 462 | 2146 | 614 |  | 2211 | N/A | 1993 | N/A | 2482 | N/A |  | 22.9 | 3.4 | 22.4 | 3.5 | 23.5 | 3.2 |
| 2008 | 6756 |  | 1883 | 567 | 1695 | 461 | 2106 | 595 |  | 2206 | N/A | 1990 | N/A | 2473 | N/A |  | 22.9 | 3.3 | 22.4 | 3.5 | 23.5 | 3.2 |
| 2009 | 6584 |  | 1876 | 567 | 1683 | 444 | 2106 | 605 |  | 2203 | N/A | 1987 | N/A | 2472 | N/A |  | 22.9 | 3.5 | 22.4 | 3.6 | 23.6 | 3.3 |
| 2010 | 6142 |  | 1859 | 562 | 1667 | 450 | 2084 | 587 |  | 2200 | N/A | 1983 | N/A | 2468 | N/A |  | 22.9 | 3.4 | 22.4 | 3.5 | 23.6 | 3.3 |
| 2011 | 5749 |  | 1849 | 562 | 1656 | 447 | 2080 | 590 |  | 2199 | N/A | 1981 | N/A | 2468 | N/A |  | 23.0 | 3.5 | 22.4 | 3.5 | 23.6 | 3.4 |
| 2012 | 21927 |  | 1889 | 560 | 1691 | 448 | 2123 | 583 |  | 2205 | N/A | 1986 | N/A | 2477 | N/A |  | 22.9 | 3.4 | 22.3 | 3.5 | 23.6 | 3.3 |
| 2013 | 5991 |  | 1890 | 564 | 1680 | 460 | 2134 | 570 |  | 2204 | N/A | 1982 | N/A | 2479 | N/A |  | 22.9 | 3.5 | 22.3 | 3.7 | 23.5 | 3.3 |
| 2014 | 5997 |  | 1878 | 576 | 1663 | 446 | 2129 | 605 |  | 2203 | N/A | 1982 | N/A | 2478 | N/A |  | 22.9 | 3.5 | 22.4 | 3.6 | 23.5 | 3.4 |
| 2015 | 5556 |  | 1901 | 573 | 1699 | 471 | 2138 | 585 |  | 2208 | N/A | 1985 | N/A | 2484 | N/A |  | 22.9 | 3.5 | 22.2 | 3.5 | 23.7 | 3.5 |
| 2016 | 18903 |  | 1881 | 527 | 1696 | 422 | 2101 | 558 |  | 2206 | N/A | 1988 | N/A | 2477 | N/A |  | 23.0 | 3.3 | 22.4 | 3.4 | 23.7 | 3.2 |
| 2017 | 5086 |  | 1919 | 579 | 1721 | 483 | 2140 | 595 |  | 2219 | N/A | 1999 | N/A | 2491 | N/A |  | 23.1 | 3.5 | 22.5 | 3.6 | 23.8 | 3.4 |
| 2018 | 5211 |  | 1936 | 587 | 1730 | 462 | 2174 | 614 |  | 2222 | N/A | 1999 | N/A | 2497 | N/A |  | 23.0 | 3.5 | 22.4 | 3.6 | 23.8 | 3.4 |
| 2019 | 4308 |  | 1914 | 592 | 1713 | 483 | 2144 | 609 |  | 2219 | N/A | 1997 | N/A | 2494 | N/A |  | 23.1 | 3.6 | 22.5 | 3.6 | 23.9 | 3.7 |

All values are shown as mean (standard deviation). N/A, not available

**Supplementary Table 5. Age-unadjusted energy intake and body mass index for USA**

| Survey  years | *n* |  | Uncalibrated energy intake (kcal/d) | | | | | |  | Calibrated energy intake (kcal/d) | | | | | |  | Body mass index (kg/m^2^) | | | | | |
| --- | --- | --- | --- | --- | --- | --- | --- | --- | --- | --- | --- | --- | --- | --- | --- | --- | --- | --- | --- | --- | --- | --- |
|  |  |  | Total | | Women | | Men | |  | Total | | Women | | Men | |  | Total | | Women | | Men | |
| 2003 | 4232 |  | 2134 | (924) | 1788 | (681) | 2464 | (1002) |  | 2422 | (367) | 2167 | (248) | 2665 | (287) |  | 28.0 | (6.1) | 28.5 | (6.8) | 27.6 | (5.4) |
| 2004 |  |  |  |  |  |  |  |  |  |  |  |  |  |  |  |  |  |  |  |  |  |  |
| 2005 | 4265 |  | 2128 | (885) | 1746 | (637) | 2480 | (934) |  | 2438 | (368) | 2170 | (251) | 2686 | (274) |  | 28.4 | (6.7) | 28.7 | (7.3) | 28.1 | (6.1) |
| 2006 |  |  |  |  |  |  |  |  |  |  |  |  |  |  |  |  |  |  |  |  |  |  |
| 2007 | 5163 |  | 2040 | (919) | 1709 | (672) | 2365 | (1009) |  | 2415 | (367) | 2165 | (250) | 2661 | (290) |  | 28.7 | (6.5) | 29.0 | (7.0) | 28.3 | (5.9) |
| 2008 |  |  |  |  |  |  |  |  |  |  |  |  |  |  |  |  |  |  |  |  |  |  |
| 2009 | 5532 |  | 2068 | (866) | 1733 | (614) | 2409 | (949) |  | 2427 | (364) | 2178 | (250) | 2680 | (277) |  | 28.9 | (6.6) | 29.2 | (7.3) | 28.6 | (5.9) |
| 2010 |  |  |  |  |  |  |  |  |  |  |  |  |  |  |  |  |  |  |  |  |  |  |
| 2011 | 4654 |  | 2090 | (861) | 1765 | (652) | 2403 | (919) |  | 2430 | (359) | 2182 | (253) | 2668 | (275) |  | 28.5 | (6.6) | 29.0 | (7.2) | 28.1 | (6.0) |
| 2012 |  |  |  |  |  |  |  |  |  |  |  |  |  |  |  |  |  |  |  |  |  |  |
| 2013 | 4962 |  | 2069 | (872) | 1775 | (677) | 2370 | (943) |  | 2423 | (358) | 2189 | (257) | 2664 | (279) |  | 28.7 | (6.8) | 29.1 | (7.4) | 28.3 | (6.0) |
| 2014 |  |  |  |  |  |  |  |  |  |  |  |  |  |  |  |  |  |  |  |  |  |  |
| 2015 | 4898 |  | 2022 | (827) | 1742 | (651) | 2312 | (889) |  | 2427 | (354) | 2200 | (262) | 2662 | (276) |  | 29.5 | (7.0) | 30.0 | (7.7) | 28.9 | (6.3) |
| 2016 |  |  |  |  |  |  |  |  |  |  |  |  |  |  |  |  |  |  |  |  |  |  |
| 2017 | 4664 |  | 2031 | (874) | 1733 | (647) | 2330 | (965) |  | 2436 | (368) | 2198 | (267) | 2675 | (294) |  | 29.6 | (7.2) | 30.1 | (7.9) | 29.2 | (6.4) |
| 2018 |  |  |  |  |  |  |  |  |  |  |  |  |  |  |  |  |  |  |  |  |  |  |

All values are shown as mean (standard deviation).

**Supplementary Table 6. Age-unadjusted energy intake and body mass index for Japan**

| Survey  years | *n* |  | Uncalibrated energy intake (kcal/d) | | | | | |  | Calibrated energy intake (kcal/d) | | | | | |  | Body mass index (kg/m^2^) | | | | | |
| --- | --- | --- | --- | --- | --- | --- | --- | --- | --- | --- | --- | --- | --- | --- | --- | --- | --- | --- | --- | --- | --- | --- |
|  |  |  | Total | | Women | | Men | |  | Total | | Women | | Men | |  | Total | | Women | | Men | |
| 1995 | 9411 |  | 2073 | (619) | 1855 | (501) | 2327 | (650) |  | 2254 | N/A | 2039 | N/A | 2522 | N/A |  | 22.7 | (3.2) | 22.5 | (3.3) | 23.0 | (3.0) |
| 1996 | 9326 |  | 2029 | (606) | 1832 | (505) | 2256 | (636) |  | 2245 | N/A | 2035 | N/A | 2504 | N/A |  | 22.7 | (3.1) | 22.5 | (3.3) | 22.9 | (3.0) |
| 1997 | 9135 |  | 2030 | (595) | 1831 | (499) | 2265 | (617) |  | 2242 | N/A | 2033 | N/A | 2506 | N/A |  | 22.7 | (3.2) | 22.5 | (3.3) | 23.0 | (3.0) |
| 1998 | 9569 |  | 2009 | (590) | 1801 | (488) | 2249 | (610) |  | 2244 | N/A | 2028 | N/A | 2510 | N/A |  | 22.9 | (3.2) | 22.6 | (3.3) | 23.3 | (3.0) |
| 1999 | 7997 |  | 1997 | (594) | 1796 | (495) | 2233 | (617) |  | 2237 | N/A | 2024 | N/A | 2505 | N/A |  | 22.8 | (3.2) | 22.5 | (3.3) | 23.2 | (3.1) |
| 2000 | 8211 |  | 1975 | (588) | 1776 | (493) | 2203 | (606) |  | 2239 | N/A | 2021 | N/A | 2496 | N/A |  | 22.8 | (3.3) | 22.5 | (3.4) | 23.2 | (3.1) |
| 2001 | 8265 |  | 1969 | (611) | 1778 | (504) | 2194 | (649) |  | 2229 | N/A | 2019 | N/A | 2497 | N/A |  | 22.9 | (3.2) | 22.5 | (3.3) | 23.4 | (3.1) |
| 2002 | 7647 |  | 1946 | (571) | 1751 | (468) | 2173 | (597) |  | 2227 | N/A | 2015 | N/A | 2493 | N/A |  | 23.0 | (3.3) | 22.6 | (3.4) | 23.5 | (3.2) |
| 2003 | 7631 |  | 1937 | (598) | 1728 | (483) | 2177 | (629) |  | 2220 | N/A | 2007 | N/A | 2489 | N/A |  | 22.9 | (3.3) | 22.6 | (3.5) | 23.3 | (3.2) |
| 2004 | 6186 |  | 1912 | (560) | 1722 | (459) | 2137 | (586) |  | 2214 | N/A | 2000 | N/A | 2482 | N/A |  | 22.9 | (3.2) | 22.4 | (3.3) | 23.4 | (3.2) |
| 2005 | 5979 |  | 1912 | (578) | 1720 | (482) | 2135 | (600) |  | 2216 | N/A | 2002 | N/A | 2481 | N/A |  | 23.0 | (3.4) | 22.6 | (3.5) | 23.5 | (3.2) |
| 2006 | 6530 |  | 1905 | (565) | 1714 | (463) | 2127 | (593) |  | 2213 | N/A | 1999 | N/A | 2480 | N/A |  | 23.0 | (3.3) | 22.6 | (3.4) | 23.5 | (3.2) |
| 2007 | 6532 |  | 1913 | (575) | 1711 | (455) | 2148 | (608) |  | 2215 | N/A | 1993 | N/A | 2485 | N/A |  | 22.9 | (3.4) | 22.3 | (3.5) | 23.6 | (3.2) |
| 2008 | 6756 |  | 1883 | (557) | 1695 | (457) | 2105 | (583) |  | 2205 | N/A | 1989 | N/A | 2471 | N/A |  | 22.9 | (3.3) | 22.4 | (3.5) | 23.5 | (3.2) |
| 2009 | 6584 |  | 1876 | (554) | 1682 | (439) | 2108 | (588) |  | 2203 | N/A | 1985 | N/A | 2474 | N/A |  | 22.9 | (3.5) | 22.4 | (3.6) | 23.6 | (3.4) |
| 2010 | 6142 |  | 1859 | (549) | 1667 | (442) | 2084 | (575) |  | 2200 | N/A | 1983 | N/A | 2468 | N/A |  | 22.9 | (3.4) | 22.4 | (3.5) | 23.6 | (3.3) |
| 2011 | 5749 |  | 1846 | (549) | 1654 | (441) | 2075 | (575) |  | 2197 | N/A | 1978 | N/A | 2464 | N/A |  | 23.0 | (3.5) | 22.4 | (3.5) | 23.6 | (3.4) |
| 2012 | 21927 |  | 1886 | (549) | 1689 | (443) | 2119 | (570) |  | 2201 | N/A | 1984 | N/A | 2474 | N/A |  | 22.9 | (3.4) | 22.3 | (3.5) | 23.5 | (3.3) |
| 2013 | 5991 |  | 1887 | (554) | 1679 | (453) | 2129 | (562) |  | 2206 | N/A | 1980 | N/A | 2474 | N/A |  | 22.9 | (3.5) | 22.4 | (3.6) | 23.5 | (3.3) |
| 2014 | 5997 |  | 1874 | (561) | 1661 | (438) | 2123 | (585) |  | 2205 | N/A | 1980 | N/A | 2471 | N/A |  | 22.9 | (3.5) | 22.5 | (3.6) | 23.5 | (3.3) |
| 2015 | 5556 |  | 1898 | (560) | 1700 | (464) | 2130 | (573) |  | 2205 | N/A | 1985 | N/A | 2478 | N/A |  | 22.9 | (3.5) | 22.3 | (3.5) | 23.7 | (3.5) |
| 2016 | 18903 |  | 1878 | (506) | 1696 | (413) | 2096 | (532) |  | 2203 | N/A | 1984 | N/A | 2470 | N/A |  | 23.0 | (3.3) | 22.4 | (3.4) | 23.7 | (3.2) |
| 2017 | 5086 |  | 1914 | (562) | 1720 | (467) | 2134 | (580) |  | 2220 | N/A | 1996 | N/A | 2483 | N/A |  | 23.1 | (3.5) | 22.6 | (3.6) | 23.8 | (3.4) |
| 2018 | 5211 |  | 1930 | (567) | 1728 | (454) | 2164 | (592) |  | 2224 | N/A | 1997 | N/A | 2489 | N/A |  | 23.1 | (3.5) | 22.5 | (3.6) | 23.8 | (3.4) |
| 2019 | 4308 |  | 1915 | (580) | 1717 | (494) | 2141 | (587) |  | 2219 | N/A | 1995 | N/A | 2486 | N/A |  | 23.2 | (3.6) | 22.5 | (3.6) | 23.9 | (3.6) |

All values are shown as mean (standard deviation). N/A, not available


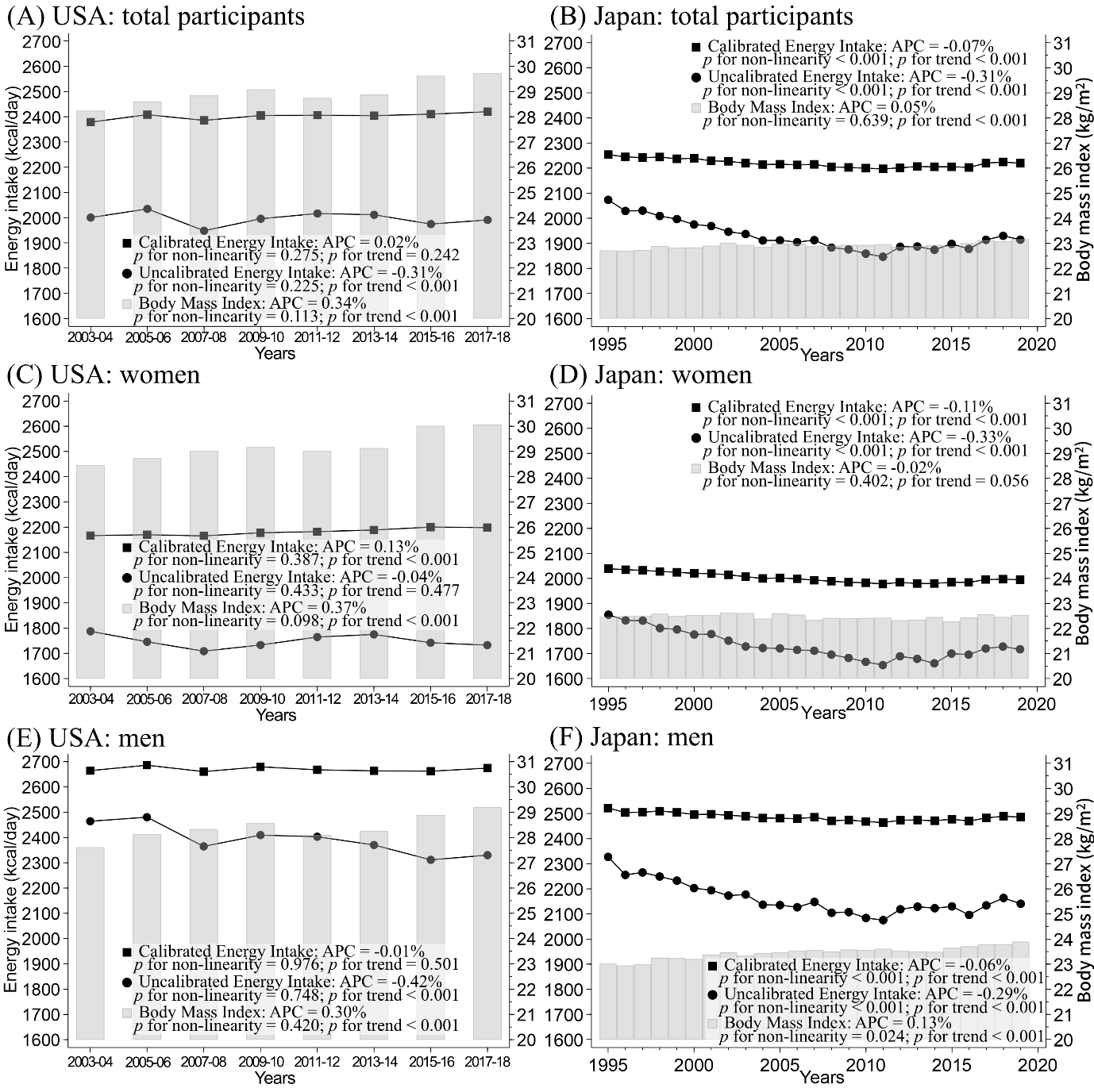


**Supplementary Figure 1.** Age-unadjusted trends in energy intake with or without biomarker-calibration, and body mass index from 2003 to 2018 in USA and from 1995 to 2019 in Japan

(A) *n* = 38,370 in total participants, (C) *n* = 19,076 in women, and (E) *n* = 19,294 in men in USA. (B) *n* = 200,629 in total participants, (D) *n* = 110,780 in women, (F) *n* = 89,849 in men in Japan. Solid lines represent mean energy intake with (■) or without (●) biomarker-calibration. The histogram shows the distribution of mean body mass index. The p-value of the linear trend was calculated by treating the exposure variable as a continuous variable. Statistical significance of non-linearity was assessed using a Wald test, comparing the likelihood ratio of the spline model with the linear model, and p-values of <0.05 were regarded as indicating a statistically significant non-linear relationship between the exposure and outcome. APC, annual percentage change


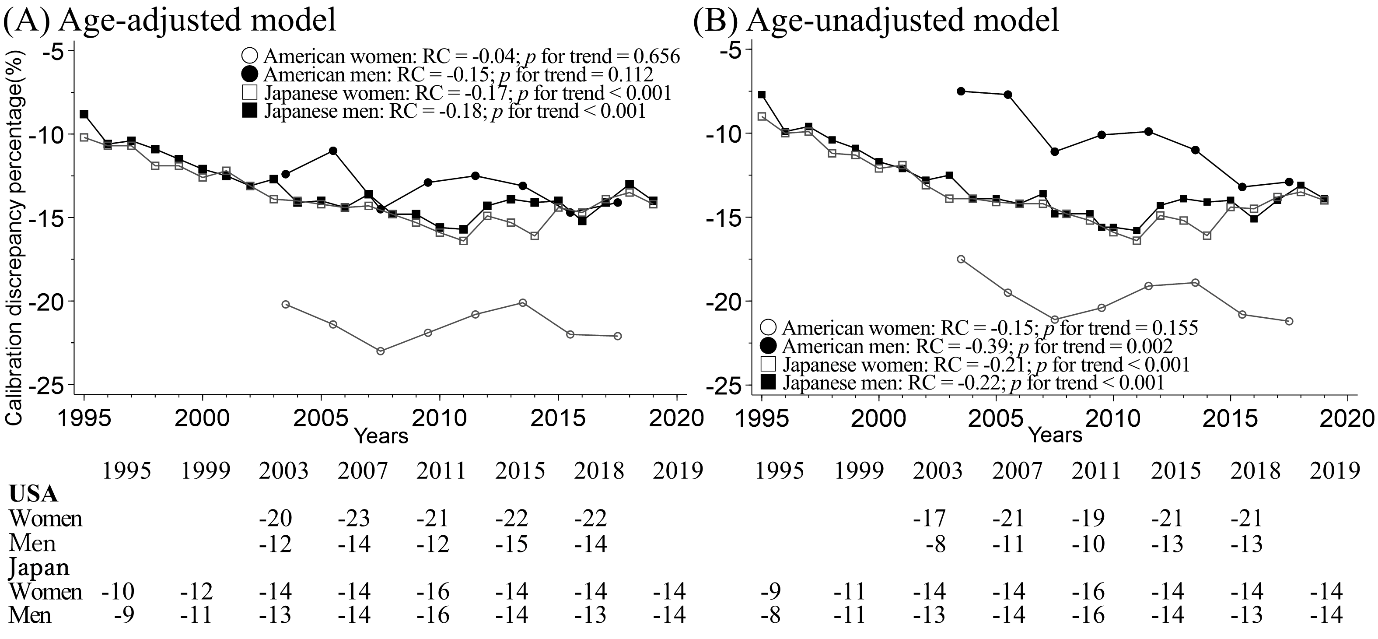
**Supplementary Figure 2**. Trends in calibration discrepancy percentage of energy intake from 2003 to 2018 in USA and from 1995 to 2019 in Japan

(A, B) *n* = 19,076 in American women, *n* = 19,294 in America men, *n* = 110,780 in Japanese women, and *n* = 89,849 in Japanese men. Solid lines represent mean calibration discrepancy of energy intake in USA (women: ○, men: ●) and Japan (women: □, men: ■). The calibration discrepancy percentage of energy intake was calculated using the following formula: (uncalibrated energy intake - calibrated energy intake) / calibrated energy intake × 100. The p-value of the linear trend was calculated by treating the exposure variable as a continuous variable. P-values of < 0.05 were regarded as indicating a statistically significant linear relationship between the exposure and outcome. All values in the age-adjusted model were calculated by regression analysis for the USA and for 2010 age groups by sex for Japan. RC, regression coefficient


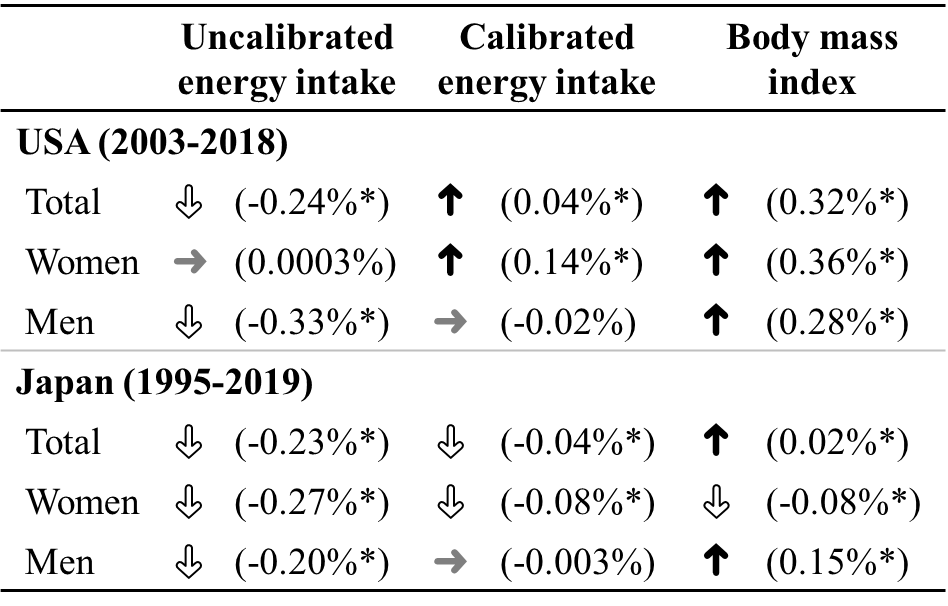


**Supplementary Figure 3.** Schematic diagram of linear regression analysis results for age-adjusted models from 2003 to 2018 in USA and from 1995 to 2019 in Japan

All values in the age-adjusted model were calculated by regression analysis for the USA. and for 2010 age groups by sex for Japan. Arrows represent the direction of significant change (annal percentage changes): increase ( ↑ ), decrease ( ↓ ), and no statistically change ( → ). **P* < 0.05
